# Public health round-up

**DOI:** 10.2471/BLT.15.010915

**Published:** 2015-09-01

**The BREATHE exhibition**

A woman cooks over a traditional wood burning stove in her home village of San Lorenzo in Guatemala. An estimated 4.3 million lives are lost prematurely each year due to household air pollution. The photograph is part of the BREATHE exhibition that was shown at the WHO headquarters in Geneva last month and will be travelling to the United Nations climate change conference in Paris, France from 30 November to 11 December.

**Figure:**
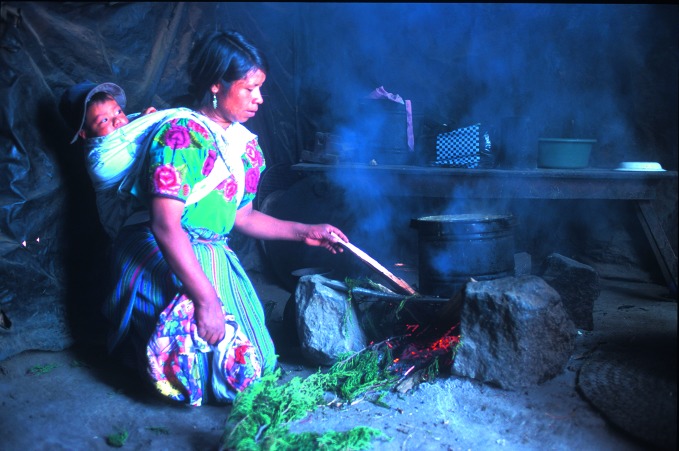
WHO/Nigel Bruce

## New development era

World leaders are set to adopt new sustainable development goals this month that aim to eradicate extreme poverty, promote prosperity and people's well-being, while protecting the environment, by 2030.

More than 150 world leaders are expected to attend the Sustainable Development Summit at the United Nations Headquarters in New York from 25 to 27 September.

Entitled “Transforming our World: The 2030 Agenda for Sustainable Development”, the final draft document outlines 17 goals comprising 169 targets and indicators.

Goal 3 focusses on health and comprises 13 targets, including ones on maternal and child health, communicable and noncommunicable diseases as well as universal health coverage and financing of health services in general.

The new sustainable development agenda builds on the eight Millennium Development Goals that helped more than 700 million people to escape poverty over the past 15 years and tackled hunger, disease, gender inequality, access to water and sanitation by 2015.

“This is the people's agenda, a plan of action for ending poverty in all its dimensions, irreversibly, everywhere, and leaving no one behind,” said United Nations Secretary-General Ban Ki-moon, welcoming the final draft document.

https://sustainabledevelopment.un.org/index.html

## Malaria vaccine

The World Health Organization (WHO) will convene a meeting of experts next month to discuss the efficacy and safety of a candidate malaria vaccine, and make recommendations to WHO on its use.

During the Strategic Advisory Group of Experts (SAGE) meeting, SAGE and the Malaria Policy Advisory Committee (MPAC), will jointly discuss malaria disease epidemiology and clinical profile, as well as the vaccine characteristics, control strategies, economic considerations, health-system opportunities and interaction with pre-existing malaria interventions.

WHO is due to make its official policy position available by the end of November.

The vaccine was developed by GlaxoSmithKline Biologicals and the PATH Malaria Vaccine Initiative with support from the Bill & Melinda Gates Foundation. It targets one of the four main forms of malaria parasite, *P. falciparum* and, if licenced, would become the first vaccine for the disease.

In April, phase 3 trial results focused on two age groups of infants: aged 6–12 weeks and 5–17 months. Vaccine efficacy against clinical malaria in those aged 5–17 months, who received four doses on a 0, 1, 2 and 20 month schedule, was 39% over the duration of the trial.

With a four-dose schedule, the overall efficacy against severe malaria in those aged 5–17 months was 31.5% with reductions in severe anaemia, malaria hospitalizations and all-cause hospitalizations also seen.

Vaccine efficacy against clinical malaria in infants aged 6–12 weeks was 27% in the group that received four doses and 18% in the group that did not receive the fourth.

In infants aged 6–12 weeks, no significant efficacy was noted against severe malaria, with or without a fourth dose.

http://www.who.int/immunization/research/development/malaria_vaccine_qa

## New Codex fund

WHO and the Food and Agriculture Organization of the United Nations (FAO) plan to set up a new fund to boost the participation of developing and transition economy countries in setting international food standards.

The United Nations organizations are seeking US$ 3.3 million in annual funding for the FAO–WHO Codex Trust Fund that is to be launched next year.

The new fund, which will run from 2016 to 2027, was endorsed last month by members of the Codex Alimentarius Commission, a global body established by the two United Nations agencies in 1963 to protect consumer health and ensure fair practices in international food trade.

The new fund builds on its predecessor that closes in December having substantially increased participation in Codex activities over the last 12 years, according to the final project evaluation.

“By funding country representatives to attend the Codex meetings, Codex Trust Fund 1 acted as a catalyst for increased participation, exposing them to the Codex process and highlighting the importance of the Codex for their countries,” said Catherine Mulholland, who manages the Codex Trust Fund at WHO.

“Codex Trust Fund 2 will take the next step by addressing the barriers that exist at national level that prevent countries from engaging effectively in Codex,” Mulholland said, citing “under-functioning of national Codex structures; lack of political and economic support for Codex; lack of understanding of Codex processes and how these can work for countries”.

These barriers will be addressed by providing tailored funding and technical assistance in a series of projects that will run for several years for specific countries or groups of countries, she said.

The Codex Alimentarius Commission is an intergovernmental body of FAO and WHO with a membership of 185 countries and one organization, the European Union. The new fund was approved at the Commission’s 38th session held at WHO headquarters in Geneva from 6 to 11 July.

http://www.who.int/foodsafety/en/

**Cover photo**

Students make their way to a shuttle boat after classes in Golachhari village, in the Rangamati district of Bangladesh. Their school is supported by the Child Friendly Schools programme of the United Nations Children’s Fund that seeks to transform the school environment into one in which children will thrive, learn and grow.

**Figure:**
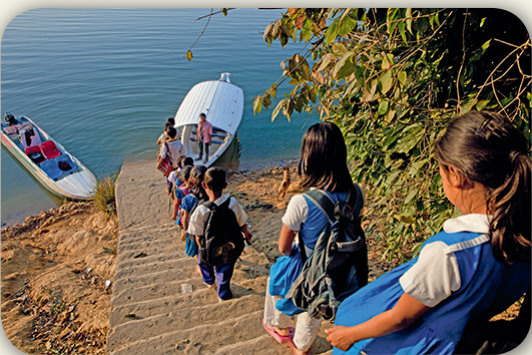
© UNICEF/BANA2014-00338/Mawa

## Safe abortion

A new WHO guideline recommends certain forms of task-shifting among the health workers who provide abortion services and post-abortion care, given the dire shortages of physicians in many countries.

An estimated 21.6 million unsafe abortions take place each year partly due to the lack of trained service providers, according to a WHO guideline entitled *Health worker roles in providing safe abortion care and post-abortion contraception*.

The guideline, which was released in July, is aimed at government policy-makers, the managers of reproductive health programmes as well as nongovernmental organizations and professional bodies involved in planning and managing abortion and post-abortion care.

The guideline provides a range of evidence-based options for involving a wide range of health-care workers in providing safe abortion care, in the management of complications of abortion and in post-abortion contraception provision.

http://www.who.int/reproductivehealth/publications/unsafe_abortion/abortion-task-shifting

## World Hepatitis Summit

The first World Hepatitis Summit will be held in Glasgow, Scotland on 2–4 September.

The summit will bring together national policy-makers, public health experts and representatives of civil society to help build global momentum towards the eventual elimination of hepatitis as a global public health threat. 

It follows governments’ commitment to tackle the disease shown in the adoption of a resolution (WHA67.R6) on viral hepatitis at the World Health Assembly in 2014. The resolution calls on countries to develop and implement national viral hepatitis strategies and for WHO to assess the feasibility of hepatitis elimination with a view to setting global targets.

http://www.who.int/hiv/events/first-hepatitis-summit-2015

## Transgender and HIV

WHO has issued its first policy brief on the prevention and treatment of HIV for the transgender population.

The policy brief entitled, *Transgender people and HIV*, is intended as a resource for governments, donors and health programme implementers to help them identify what needs to be done to address HIV among transgender people.

Transgender people are those that feel that their gender is different from the sex they were assigned at birth. They are one of several groups that are highly vulnerable to HIV infection, according to the 2014 *WHO Consolidated guidelines on HIV prevention, diagnosis, treatment and care*.

While data is limited due to lack of population size estimates and issues with stigma and discrimination, the policy brief found that transgender women, for example, had a risk of HIV infection that was 49 times greater than in the general population.

http://www.who.int/hiv/pub/transgender/transgender-hiv-policy

**Looking ahead**

25–27 September – United Nations Summit to adopt the post-2015 development agenda. New York, United States of America.

11–12 October – World Health Summit of M8 Alliance of Academic Health Centers, Universities and National Academies. Berlin, Germany

30 November–11 December – United Nations Climate Change Conference in Paris, France.

